# The Nutritional Quality of Organic and Conventional Food Products Sold in Italy: Results from the Food Labelling of Italian Products (FLIP) Study

**DOI:** 10.3390/nu12051273

**Published:** 2020-04-30

**Authors:** Margherita Dall’Asta, Donato Angelino, Nicoletta Pellegrini, Daniela Martini

**Affiliations:** 1 Department of Animal Science, Food and Nutrition, Università Cattolica del Sacro Cuore, 29122 Piacenza, Italy; margherita.dallasta@unicatt.it; 2 Faculty of Bioscience and Technology for Food, Agriculture and Environment, University of Teramo, 64100 Teramo, Italy; dangelino@unite.it; 3 Department of Agricultural, Food, Environmental and Animal Sciences, University of Udine, 33100 Udine, Italy; 4 Department of Food, Environmental and Nutritional Sciences (DeFENS), Università degli Studi di Milano, 20133 Milan, Italy; daniela.martini@unimi.it

**Keywords:** organic food, food labeling, nutrition facts, nutritional quality

## Abstract

The market for organic products is growing rapidly, probably attributable to the general customer perception that they are healthier foods, with a better nutritional profile than conventional ones. Despite this, the available studies show limited differences in the nutrient profile of organically and conventionally primary food products. Apart from this literature, no studies have focused on the nutrition profile of commercially prepacked foods. Thus, the aim of the present survey was to compare the nutritional quality intended as nutrition facts of organic and conventional prepacked foods sold in Italy. A total of 569 pairs of prepacked products (organic and their conventional counterparts) were selected from nine food categories sold by online retailers. By comparing organic and conventional products in the “pasta, rice and other cereals” category, the former were lower in energy, protein, and higher in saturates compared to the latter. Organic “jams, chocolate spreads and honey” products were lower in energy, carbohydrates, sugars and higher in protein than their regular counterparts. No differences were found for energy, macronutrients and salt for other categories. Therefore, based on the mandatory information printed on their packaging, prepacked organic products are not of a superior nutritional quality than conventional ones, with just a few exceptions. Consequently, the present study suggests that organic certification cannot be considered an indication of better overall nutritional quality. Further studies examining the nutritional quality of organic foods, taking into account the ingredients used, might better explain the results obtained.

## 1. Introduction

Organic production has become increasingly important worldwide, as a potential alternative to conventional intensive agriculture, due to great concerns about the environment, food safety, and human health [1,2,3]. In developed countries, demand for organic products is steadily rising and a relevant proportion of food consumed comes from organic sources [4]. Undoubtedly, the increase in production and consumption of organic foods is one of the major market trends of recent years [5]. Following the U.S., in 2017, the European organic food market was the second largest in the world in terms of sales with most of the organic food retailers mainly located in Germany, France and Italy [5]. In particular, a recent survey performed in Italy confirmed that the value of sales of the organic foods follows a positive trend [6]. 

As stated in Council Regulation (EC) No 834/2007 [7], organic products can be defined as food products deriving from “organic production”, which means the use of the production method compliant with the rules established in the European Regulation, at all stages of production, preparation and distribution. Organic production must be based on the appropriate design and management of biological processes based on ecological systems using natural resources while restricting the use of external inputs [7].

Considering the definition and the principles of organic production, the consumption of organic foods may reduce exposure to nitrate and pesticide residues due to the strict limitation of the use of chemically synthesized inputs [3,8]. However, the significance of these differences is questionable, because actual levels of contamination in organic and conventional foods are generally well below acceptable limits [9] and in general the proximity to sources of contamination (e.g., traffic, chemical industries) seems to have a crucial role in the occurrence of environmental pollutants in foodstuffs [10]. This may partially explain why evidence on the association between the consumption of organic foods and the risk of developing chronic diseases is generally weak [11,12].

Conversely, the definition given by Council Regulation (EC) No 834/2007 [7] does not include any mention of possible differences in terms of the nutritional quality and healthiness of organic and conventional products. Despite this, evidence suggests that people tend to perceive organic foods as healthier than standard, non-organic foods [13,14,15], with healthiness primarily understood as nutritional value [16,17]. This is probably due to the so called “health halo effect”, which induces the consumer to overestimate the healthfulness of a food with a specific attribute [18]. For instance, compared to conventional products, organic foods are perceived as of lower calorific value and more palatable to consumers who also generally show more intention to pay for organic products [13,15,19,20,21]. 

In this scenario, several studies have investigated the possible differences between several categories of organic and conventional foods [22,23], including fruit and vegetables [24,25], meat [26], milk [27] and dairy foods [28]. Only small differences in nutrient content between organic and conventional products have been evidenced, mostly related to differences in production methods [22]. For instance, different agricultural managements have been shown to play a role on the polyphenol content in vegetables, plausibly because a higher polyphenolic content is observed when less nitrogen fertilizer is added to the soil [29]. However, no conclusive data have clearly evidenced neither a higher nutritional quality of organic products when compared with the conventional alternatives [22,30], nor nutrition-related health effects for organic products [31]. However, most of the studies have been focused on the nutritional quality of primary products. Conversely, the possible differences between processed and prepacked organic and conventional products have been barely investigated.

Based on these premises, the aim of the present study was to investigate and compare the nutritional quality of pairs of organic and conventional prepacked foods currently sold in Italy with the same brand name, by collecting the nutritional data on their packaging. This study was performed as part of the Food Labelling of Italian Products (FLIP) Study that aims at systematically investigating the overall quality of prepacked foods sold on the Italian market. 

## 2. Materials and Methods 

### 2.1. Data Collection

The online search for information was conducted from January 2019 until July 2019 on the home-shopping website of the major retailers present on the Italian market (Auchan, Bennet, Carrefour, Conad, Coop Italia, Crai, Despar, Esselunga, Il Gigante, Iper, Pam Panorama, Selex, Sidis). 

The systematic search was performed by specifically focusing on the selection of all potential pairs of products (organic and conventional) of the same brand considered if available in at least one online shop. We included all the prepacked foods (regardless of the level of processing) for which, as stated in the Council Regulation (EC) no. 1169/2011 [32], mandatory food information shall appear directly on the package or on a label attached thereto. Products were considered eligible as organic food if the Community logo referred to in Article 25(1) Regulation 834/2007 [7] as regards pre-packaged food was present on the packaging. 

The exclusion criteria for product selection were: (i) not prepacked; (ii) organic foods with no conventional counterparts of the same brand; (iii) incomplete images of all the sides of the pack; (iv) unclear images of nutrition declaration or list of ingredients; (v) nine products that were marked as “product currently unavailable” in all the online stores which were selected throughout the data collection period.

### 2.2. Data Extraction 

Data from the complete images of all the sides of the pack were collected for all the selected products. For each food item, as previously described [33], the quali-quantitative and specifically regulated (mandatory) information was documented: company name, brand name, descriptive name, energy (kcal/100 g or 100 mL), total fat (g/100 g or 100 mL), saturates (g/100 g or 100 mL), carbohydrate (g/100 g or 100 mL), sugars (g/100 g or 100 mL), protein (g/100 g or 100 mL), and salt (g/100 g or 100 mL). Moreover, the number of nutrition claims (NC) as listed in the Council Regulation (EC) No 1924/2006 [34] was collected.

Data were extracted once (by DM) but the accuracy of the extracted data was double-checked by two researchers (MDA, DA) and inaccuracies were resolved through secondary extractions made by a third researcher (DM). 

A dataset was created with all the collected data and items were sub-grouped for specific comparisons by considering the descriptive name reported and the presence/absence of organic declaration. Based on the descriptive name, the food items were classified in the following categories: (i) sweet cereal-based foods; (ii) bread and substitutes; (iii) pasta, rice and other cereals; (iv) milk, dairy foods and plant based-drinks; (v) fruit juices, nectars and iced teas; (vi) jams, chocolate spreads and honey; (vii) fruit and vegetable-based foods; (viii) legumes; (ix) oils, fats and dressings. 

### 2.3. Statistical Analysis

Statistical analysis was carried out using IBM SPSS Statistics^®^ (Version 25.0, IBM corp., Chicago, IL, USA) and performed at *p* < 0.05 of significance level. The normality of data distribution was firstly verified through the Kolmogorov-Smirnov test and rejected. Therefore, variables were expressed as median (interquartile range). Data of energy and nutrient contents per 100 grams or 100 mL of products for each item were analyzed with the Mann-Whitney non-parametric test for two independent samples (for differences between organic and conventional products).

## 3. Results

### 3.1. Number and Categories of Products

A total of 569 pairs of products were selected through the online store search. The number and the type of pair items for each category is reported in Table 1. The largest category was “milk, dairy foods and plant based-drinks” (*n* = 123), of which 74% of the items were yogurts and cheese. The second largest category was “pasta, rice and other cereals” one (*n* = 104 pairs), while the smallest ones were “bread and substitutes” (*n* = 42) and “sweet cereal based-foods” (*n* = 28). The number of organic and conventional products with at least one NC was also analyzed. Overall, the number of items with an NC was relatively similar for all categories of organic and their conventional counterparts, except for “pasta, rice and other cereals”, which had a greater number of products with claims for organic products (*n* = 60) than for non-organic ones (*n* = 7).

### 3.2. Nutritional Comparison Among Organic and Conventional Products

**Figure 1:**
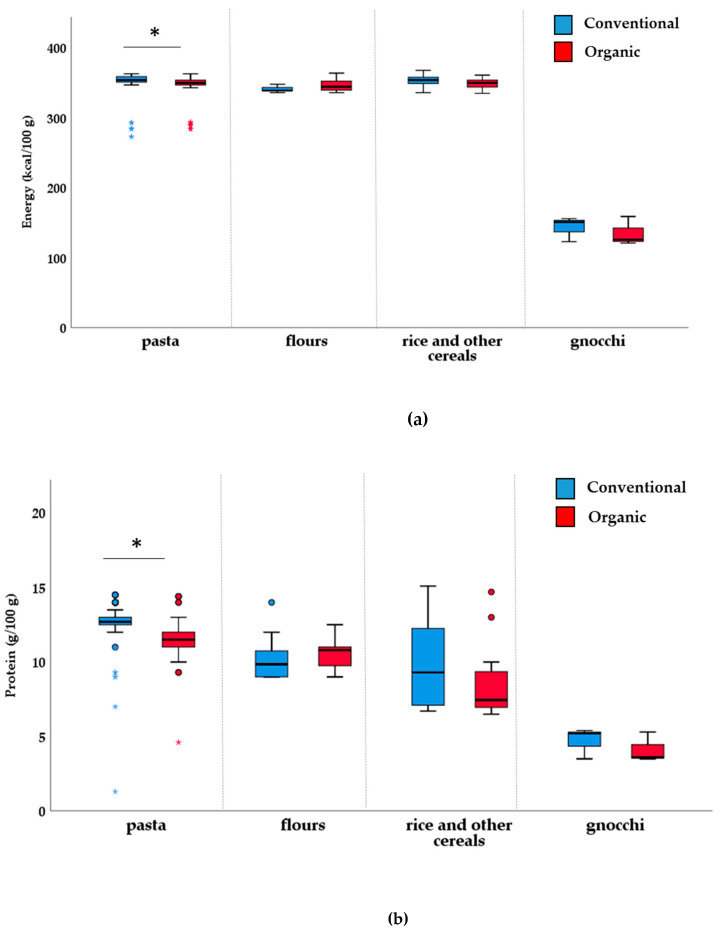
Box plot for energy (kcal/100 g) (**a**) and protein (g/100 g) (**b**) of “pasta, rice and other cereals” category. * *p* < 0.05, Mann–Whitney non-parametric test for two independent samples.

**Figure 2:**
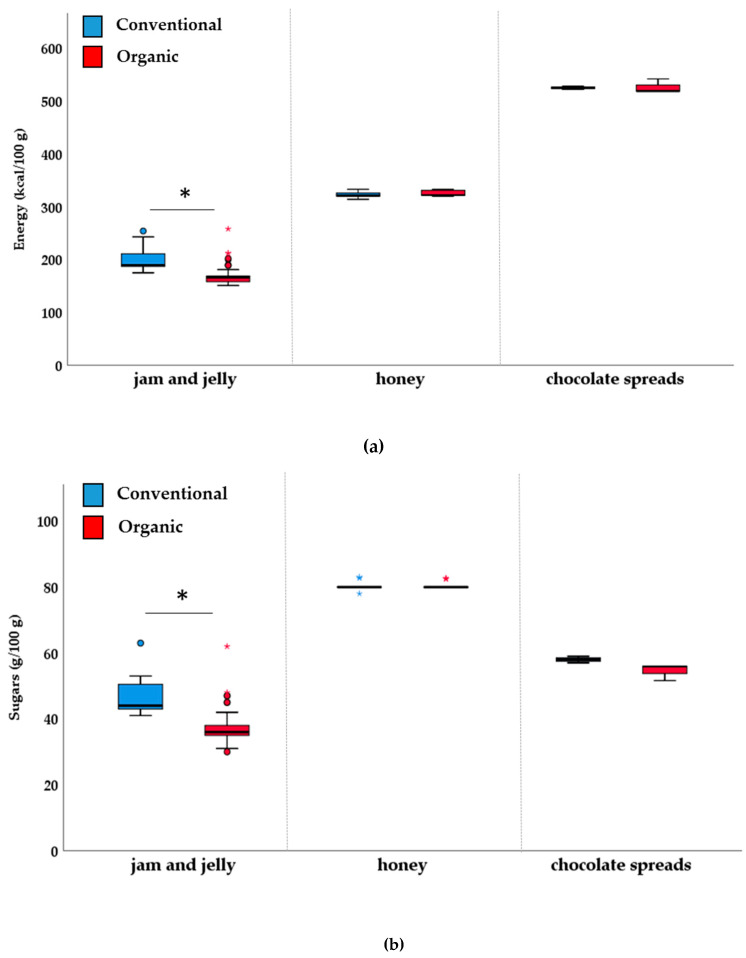
Box plot for energy (kcal/100 g) (**a**) and sugars (g/100 g) (**b**) of “jams, chocolate spreads and honey” category. * *p* < 0.05, Mann–Whitney non-parametric test for two independent samples.

The mandatory nutrition information indicated by Council Regulation (EU) no. 1169/2011 [32], across the considered categories of products, is reported in Table 2. Overall, results showed that there are slight differences in terms of nutritional quality between the organic and their conventional counterparts, with only significant differences for two out of the nine food categories investigated. Concerning the “pasta, rice and other cereals” category, the data show a slightly, but significant, lower median of energy of organic products (*p* < 0.001) compared to the conventional ones. These results may be explained by the significantly higher median protein content in this conventional food category compared to the organic one. Conversely, in the organic “pasta, rice and other cereals” category, a significantly higher saturates median value was found compared to conventional products (*p* = 0.007). More appreciable differences were found for the category “jams, chocolate spreads and honey”, where organic products, compared with their conventional counterparts, were characterized by a lower (*p* < 0.001) median of energy, total carbohydrates and sugars. On the contrary, in this category, a significantly higher median protein content was found in organic products compared conventional ones (*p* = 0.002). The statistical significant differences observed in the categories “pasta, rice and other cereals” and “jams, chocolate spreads and honey” may be attributable to significant differences in the subcategories “pasta” (Figure 1) and “jam and jelly” (Figure 2), respectively. No other differences were found for other categories, including “Sweet cereal based-foods”, “Bread and substitutes”, “Milk, dairy foods and plant-based drinks”, “Fruit juices, nectars and iced teas”, “Fruit and vegetable-based products, “Legumes”, and “Oils, fats and dressings”.

To better evaluate the nutritional quality of both organic and non-organic paired products within the different categories, data related to the single types belonging to the nine categories are reported in Supplementary Table S1. Organic pasta products presented significantly lower median energy (*p* < 0.001) and significantly lower amounts of carbohydrates (*p* = 0.001) than in conventional pasta. Moreover, saturates were present in significantly lower amounts in non-organic products than organic ones (*p* = 0.003). Protein displayed significantly higher in non-organic pasta than organic pasta products (*p* < 0.001); on the contrary, a lower median salt amount was found in organic pasta compared to non-organic ones (*p* = 0.049). By analyzing the type of nutrition claim for “pasta” subcategory, 53 organic items out of the 77 pairs of products presented the claim related to fiber content, while only two items were present in regular counterparts. Within the same category of products, organic flours differed from the regular ones only for the higher amount of sugar content (*p* = 0.012).

Organic jam and jelly presented a significantly lower median value energy than conventional products (*p* < 0.001). Total carbohydrates, almost totally represented by sugars, were significantly higher in non-organic jam and jelly items compared to organic ones (*p* < 0.001 as for total carbohydrates and sugars). Finally, protein were lower in conventional products than in organic counterparts (*p* = 0.011). 

Among bread and substitutes, organic wraps were higher in sugars (*p* = 0.010), but lower in salt (*p* = 0.002) than conventional ones. On the contrary, organic crackers were lower in sugars than non-organic ones (*p* = 0.023). 

Organic yogurts were slightly higher in salt than conventional ones (*p* < 0.001). Interestingly, organic plant based-drinks had significantly lower median energy values than conventional ones (*p* = 0.016) mainly attributable to their significantly lower amount total carbohydrates (*p* = 0.008) and sugars (*p* = 0.016) than non-organic products. 

## 4. Discussion

To our knowledge, this is the first study to provide a snapshot of the nutritional quality of organic prepacked products sold on the home-shopping website of the major retailers present on the Italian market, by selecting pairs of products (organic and conventional ones) of the same brand present in at least one shop and considering the legally required data regarding nutritional content, printed on the packaging. There are two main reasons why only organic products for which a non-organic counterpart is available from the same brand were chosen. Firstly, although this inclusion criterion might have lowered the number of products screened, it avoids the weakness of the statistical comparison of data from categories with substantial differences in terms of number of organic and non-organic items. Furthermore, this approach may allow us to take into account the relevant role of a brand in customers’ intention-to-buy. In fact, it has been ascertained that consumers tend to perceive the brand as “a first sign of quality” and then to consider other evaluation criteria [35].

The results obtained from 569 pairs of items indicated an overall comparable nutritional profile of the two alternatives. Only two out of the nine analyzed categories presented statistically significant differences, based on both energy and nutritional content. Organic “pasta, rice and other cereals” had a lower energy density and protein content in 100 g of the product than the conventional alternatives. Moreover, a higher amount of saturates was evidenced for organic products than regular ones within such a category, even if this difference can be considered of marginal nutritional significance due to the generally low amount of saturates in these types of products. Within this category, the subcategory “pasta” had most likely contributed to these significant differences. This might be due to the fact that organic pasta had a higher number of products with a nutrition claim related to the fiber content compared to conventional ones and this may in turn influence the content of the other analyzed nutrients. Furthermore, the lower energy, carbohydrate and sugar contents observed in organic “jams, chocolate spreads and honey” may be attributed to significant nutrient changes observed in the subcategory “jam and jelly”. In this case, differences cannot be explained by the presence of several nutrition claims, because no claims were found neither among organic nor among conventional items.

Although no significant differences were found for other food categories, further analysis of subcategories showed some interesting differences. The most remarkable difference was the higher sugar content in conventional plant-based drinks compared to organic alternatives. However, on the whole, organic foods do not seem to be of a higher nutritional quality than conventional foods from the same brand.

The focus of this research was to investigate the nutritional quality of products, which is not only the result of the organic production methods applied in the food chain, but depends also on the ingredients selected for the product formulation. For this reason, a comparison of the results obtained in the present survey with previous studies appears tricky, since the majority of the studies focused on components that were not considered in the present survey. Indeed, previous literature suggested that there were differences in the nutritional profile of organic products and non-organic ones, particularly for polyunsaturated fatty acids, vitamins, minerals, and bioactive compounds, such as polyphenols [23,27,36,37,38,39], although the information available is not exhaustive. Therefore, it is not to be excluded that the products considered in the present survey could present different amounts of some components, i.e., bioactive compounds or micronutrients, but this was out of the scope of this study, which only focused on the mandatory information to be included in the nutrition declaration compliant to Regulation (EC) 1169/2011 [32]. 

Only few studies reported the nutritional quality of organic prepacked foods compared to conventional counterparts. For instance, sugar content in German breakfast cereals, both generic and specific for children, was significantly lower in organic products than in the conventional ones [40]. In contrast, another study conducted in the U.S. investigating the nutritional quality of ready-to-eat breakfast cereals did not support the higher nutritional value of the organic products in comparison with conventional ones, when taking into account macronutrients and micronutrients and bioactive compounds [41]. Interestingly, in a study performed in the UK, organic yogurts were compared to all the other types of yogurts, and they showed to be higher in sugar content compared to the other types, except for desserts [42]. Finally, a study analyzing the front-of-pack declarations and the critical nutrients (free sugar, total fats, saturated fats, trans fat, and sodium) of a wide range of products sold in Brazil showed that products presenting claims related to environment, such as “organic”, were more unlikely to present high content of such nutrients [43]. 

Therefore, the evidence to date cannot support the view that organic products have a higher nutritional quality than conventional ones, also because studies concerning the health effects of organic foods in humans are still scarce and sometimes inconclusive [2,11,44,45,46]. Apart from the direct effects attributed to organic products, it is noteworthy that other factors may play a role in defining the beneficial effect of consuming organic products. Interestingly, there is evidence that people who purchase organic foods generally have a healthier lifestyle, including a healthier diet, than those who don’t buy them, thus potentially reducing the risk of several major diseases, independently of the potential additional health effects brought by organic foods [47,48,49]. Indeed, comparisons of diets containing organic and non-organic foods have been challenged because consumers of organic food most frequently maintain a healthy lifestyle [2]. For these reasons, there is still insufficient evidence to recommend organic over conventional products [25], at least from a nutritional point of view.

This study has some limitations worth highlighting. The first is in regards to the methodology of product selection, as it did not include other retail outlets, such as discount warehouses, which would be worthy of future investigation. Secondly, the present study focused on the evaluation of nutritional quality based only on mandatory information, which does not include other nutritional components, such as fiber, vitamins, minerals and bioactive compounds. Moreover, it is important to note that, considering the Regulation 1169/2011 [32], nutrition declaration can be formulated either from direct analysis of food or from data extrapolated from reference databases of food composition, which do not take into account potential differences between organic and non-organic ingredients. Another limitation is the low number of pairs for some sub-categories that may have limited the statistical significance of some results. Finally, some of the major brands of either organic or conventional products do not produce the counterparts and therefore have not been considered in the present survey. Thus, comparing the nutritional quality of all organic foods to all conventional foods would provide a valuable addition to the findings of the present study. However, it is worth remarking that the aim of the study was to compare items of the same brand, which can be considered a strength of the study. In this way, the brand name cannot act as a possible cause of bias in the results because it represents one of the most relevant factors driving the consumer’s intention to buy at the time of purchase. Another strength of the present work belongs to the fact that the studies covered several categories and subcategories of products, leading to a high number of pairs selected for the study. Moreover, collecting information from online shops results the best way to ensure that the study covers the majority of products sold by the same brand, for which both the conventional and organic alternatives exist.

The present work was conceived in the frame of the Food Labelling of Italian Products (FLIP) project, which primarily focused on systematically evaluating the nutritional quality of commercial foods sold on the Italian market, and specifically took into account the different declarations present on the packaging. As for the other declarations present on the packaging (e.g., nutrition and health claims), the presence of organic certification on the food pack seems to not be indicative of the overall quality of the products. 

## 5. Conclusions

Based on the mandatory information reported on the packaging, this original survey indicated that, with few exceptions, organic labelled prepacked products sold in Italy were not characterized by a better nutritional profile than conventional ones. Consequently, the “organic” claim should not be interpreted by consumers as proxy of “healthier” food than regular food. However, future studies are needed to broaden the analysis to other food groups not considered within the present survey. Certainly, there is the need to better investigate the nutritional quality of the single ingredients (i.e., types and amount) used for the formulation of organic products, which might sometimes be formulated taking into account the healthier perception of organic products by consumers. Moreover, further research could be aimed at analyzing data by considering the type of producers of organic/non-organic products (i.e., transnational food companies versus small companies).

## Figures and Tables

**Table 1:** Categories, total number of pairs, subcategories of products considered within each category and number of organic and conventional products with at least a Nutrition Claim (NC).

Category	Number of Pairs	Subcategories (Number of Pairs)	Number of Products with at Least a NC (Organic/Conventional)
Sweet cereal based-foods	28	Cookies (13), breakfast cereals (9), snacks (6)	8/13
Bread and substitutes	42	Wraps (11), crackers (10), breadsticks (9), bread (6), rusks (6)	12/10
Pasta, rice and other cereals	104	Pasta (77), rice and other cereals (12), wheat flour (12), gnocchi (3)	60/7
Milk, dairy foods and plant-based drinks	123	Yogurt (46), cheese (45), milk (27), plant-based drinks (5)	19/22
Fruit juices, nectars and iced teas	54	Fruit juices and nectars (52), iced teas (2)	7/12
Jams, chocolate spreads and honey	61	Jam and jelly (44), honey (14), chocolate spreads (3)	0/0
Fruit and vegetable-based foods	51	Tomato-based sauces (21), dried fruit (15), frozen vegetables (15)	6/2
Legumes	55	Dry legumes (21), canned and frozen legumes (34)	12/16
Oils, fats and dressings	51	Olive oil, other vegetable oils (32), animal fats and margarine (10), vinegar (9)	12/14

**Table 2:** Energy, macronutrients, and salt across the considered categories.

Category		Number of Items	Energy kcal/100 g or 100 mL	Total Fat g/100 g or 100 mL	Saturates g/100 g or 100 mL	Total Carbohydrates g/100 g or 100 mL	Sugars g/100 g or 100 mL	Protein g/100 g or 100 mL	Salt g/100 g or 100 mL
**Sweet cereal based-foods**	Total	56	419 (377–455)	14.0 (2.4–17.0)	2.3 (0.8–4.1)	65.5 (61.9–76.9)	21. 3(17.0–30.0)	7.8 (6.8–8.5)	0.6 (0.5–0.8)
	Conventional	28	419 (375–453)	14.0 (2.4–16.9)	2.2 (0.6–3.5)	67.4 (63.0–76.9)	23.0 (19.5–31.0)	7.3 (6.5–8.0)	0.6 (0.5–0.8)
	Organic	28	421 (380–455)	14.0 (2.4–17.0)	2.3 (1.1–5.1)	64.6 (60.5–78.3)	21.0 (16.5–27.4)	8.0 (7.2–8.8)	0.6 (0.3–0.9)
**Bread and substitutes**	Total	84	401 (305–427)	9.4 (6.2–12.0)	1.5 (0.8–2.0)	61. 7(47.1–68.0)	2.0 (1.6–3.5)	10. 7(8.2–12.3)	1. 6(1.3–1.9)
	Conventional	42	408 (303–429)	8.6 (6.5–11.5)	1.4 (1.0–2.4)	63.1 (47.1–69.9)	2.0 (1.5–4.0)	10.0 (8.1–12.0)	1.7 (1.3–1.8)
	Organic	42	395 (306–426)	9.6 (5.8–12.0)	1.7 (0.8–2.0)	59.5 (46.0–67.0)	2.0 (1.6–3.0)	11.4 (8.5–12.5)	1.5 (1.3–2.0)
**Pasta, rice and other cereals**	Total	208	351 (346–356)	1.5 (1.3–1.7)	0.3 (0.3–0.5)	71.0 (67.5–73.0)	2.9 (1.6–3.2)	12.0 (11.0–12.7)	0.0 (0.0–0.0)
	Conventional	104	354 (348–358)a	1.5 (1.3–1.5)	0.3 (0.3–0.4)b	71.5 (69.2–73.1)	2.9 (1.4–3.2)	12.5 (11.0–13.0)a	0.0 (0.0–0.0)
	Organic	104	350 (346–354)b	1.5 (1.2–1.9)	0.3 (0.3–0.5)a	71.0 (66.6–72.5)	2.9 (1.7–3.2)	11.0 (11.0–12.0)b	0.0 (0.0–0.0)
**Milk, dairy foods and plant-based drinks**	Total	246	100 (65–238)	3.7 (3.0–19.0)	2.6 (2.0–13.0)	4.8 (2.1–5.6)	4.6 (1.7–5.1)	3.7 (3.3–12.0)	0.1 (0.1–0.6)
	Conventional	123	100 (65–236)	3.6 (3.0–19.0)	2.6 (2.0–13.0)	4.9 (2.3–5.6)	4.8 (1.7–5.1)	3.7 (3.3–12.0)	0.1 (0.1–0.6)
	Organic	123	101 (64–240)	3.7 (2.3–19.0)	2.6 (1.2–13.0)	4.8 (2.1–5.6)	4.5 (1.7–5.1)	3.8 (3.2–13.0)	0.1 (0.1–0.6)
**Fruit juices, nectars and iced teas**	Total	108	57 (49–59)	0.0 (0.0–0.0)	0.0 (0.0–0.0)	13.3 (11.4–14.0)	13.0 (10.2–14.0)	0.2 (0.0–0.4)	0.1 (0.0–0.1)
	Conventional	54	57 (51–60)	0.0 (0.0–0.0)	0.0 (0.0–0.0)	13.7 (11.6–14.2)	13.2 (10.2–14.0)	0.2 (0.0–0.4)	0.0 (0.0–0.0)
	Organic	54	56 (47–59)	0.0 (0.0–0.0)	0.0 (0.0–0.0)	13.1 (11.2–14.0)	13.0 (11.2–13.7)	0.2 (0.1–0.4)	0.0 (0.0–0.0)
**Jams, chocolate spreads and honey**	Total	122	190 (168–320)	0.0 (0.0–0.2)	0.0 (0.0–0.0)	45.0 (40.0–60.0)	44.0 (37.0–59.0)	0.4 (0.0–0.6)	0.1 (0.0–0.1)
	Conventional	61	206 (187–320)a	0.0 (0.0–0.1)	0.0 (0.0–0.0)	50.0 (45.0–60.0)a	50.0 (43.0–59.0)a	0.4 (0.0–0.5)b	0.1 (0.0–0.1)
	Organic	61	168 (160–320)b	0.0 (0.0–0.3)	0.0 (0.0–0.0)	40.0 (38.0–59.0)b	37.0 (35.0–56.0)b	0.5 (0.3–0.6)a	0.1 (0.0–0.1)
**Fruit and vegetable-based products**	Total	102	54 (30–352)	0.5 (0.1–32)	0.1 (0.0–3.8)	6.0 (4.2–8.8)	3.8 (2.2–4.5)	2.5 (1.4–4.8)	0.1 (0.0–0.3)
	Conventional	51	53 (30–352)	0.5 (0.1–32.0)	0.1 (0.0–3.8)	6.0 (4.1–9.2)	3.9 (2.4–4.5)	2.7 (1.4–4.9)	0.1 (0.0–0.4)
	Organic	51	55 (30–429)	0.5 (0.1–41.0)	0.1 (0.0–4.6)	6.0 (4.2–8.7)	3.7 (1.6–4.5)	2.4 (1.3–4.7)	0.0 (0.0–0.3)
**Legumes**	Total	110	106 (87–310)	1.0 (0.5–1.9)	0.1 (0.1–0.3)	15.4 (12.2–44.0)	1.1 (0.6–2.6)	6.9 (5.5–20.1)	0.6 (0.0–0.8)
	Conventional	55	124 (89–323)	1.0 (0.5–1.9)	0.2 (0.1–0.3)	16.2 (13.2–44.8)	0.8 (0.6–2.6)	7.0 (5.6–20.9)	0.7 (0.0–1.0)
	Organic	55	97 (87–304)	0.9 (0.4–2.0)	0.1 (0.1–0.3)	14.2 (12.0–42.8)	1.2 (0.5–2.5)	6.9 (5.6–20.8)	0.6 (0.0–0.8)
**Oils, fats and dressings**	Total	102	822 (747–824)	91.5 (83.0–91.8)	13.7 (11.0–15.2)	0.0 (0.0–0.1)	0.0 (0.0–0.1)	0.0 (0.0–0.1)	0.0 (0.0–0.0)
	Conventional	51	822 (751–824)	91.3 (83.0–92.0)	13.5 (11.0–15.2)	0.0 (0.0–0.1)	0.0 (0.0–0.1)	0.0 (0.0–0.1)	0.0 (0.0–0.0)
	Organic	51	823 (739–824)	91.6 (82.0–91.8)	14.0 (11.0–15.2)	0.0 (0.0–0.1)	0.0 (0.0–0.1)	0.0 (0.0–0.1)	0.0 (0.0–0.0)

Values are expressed as median (25th–75th percentile). For each category, different letters in the same column indicate significant differences among conventional and organic products (Mann–Whitney non-parametric test for two independent samples), *p* < 0.05.

## Supplementary Materials

The following are available online at https://www.mdpi.com/2072-6643/12/5/1273/s1: Table S1: Energy, macronutrients, and salt for all the types of products analyzed.

## Finally

In this context, there remains a strong need to define the real impact of organic production on the nutritional quality of ingredients with well-designed studies, taking also into account the potential impact of the formulation of processed foods on nutrient quality and quantity.

## Appendix A

**Table A1:** SINU Young Working Group.

Marika Dello Russo	Institute of Food Sciences, National Research Council, Avellino, Italy
Stefania Moccia	Institute of Food Sciences, National Research Council, Avellino, Italy
Daniele Nucci	Veneto Institute of Oncology IOV-IRCCS, Padova, Italy
Gaetana Paolella	Department of Chemistry and Biology A. Zambelli, University of Salerno, Fisciano, Italy
Veronica Pignone	Department of Epidemiology and Prevention, IRCCS Neuromed, Pozzilli, Italy
Alice Rosi	Department of Food and Drug, University of Parma, Parma, Italy
Emilia Ruggiero	Department of Epidemiology and Prevention, IRCCS Neuromed, Pozzilli, Italy
Carmela Spagnuolo	Institute of Food Sciences, National Research Council, Avellino, Italy
Giorgia Vici	University of Camerino, Camerino, Italy
