# Supplementary material for: The Nutritional Quality of Organic and Conventional Food Products Sold in Italy: Results from the Food Labelling of Italian Products (FLIP) Study
Source: Nutrients. 2020 Apr 30;12(5):1273. doi: 10.3390/nu12051273 (PMC7282013; doi:10.3390/nu12051273)
Supplement: Supplementary file 1 [file nutrients-12-01273-s001.pdf]

Supplementary Table 1: Energy, macronutrients, and salt across the considered subcategories of products.

Subcategory		Number of Items	Energy (kcal/100 g or 100 mL)	Total fats (g/100 g or 100 mL)	Saturates (g/100 g or 100 mL)	Total Carbohydrates (g/100 g or 100 mL)	Sugars (g/100 g or 100 mL)	Protein (g/100 g or 100 mL)	Salt (g/100 g or 100 mL)
Sweet cereal based-foods									
**Cookies**	Conventional	13	449 (427–460)	16.0 (10.8–18.0)	2.2 (1.7–3.4)	68.8 (63.1–76.0)	23.0 (21.0–24.0)	8.0 (7.1–8.9)	0.7 (0.6–0.8)
	Organic	13	453 (439–470)	16.0 (11.0–19.7)	2.4 (2.0–6.9)	65.0 (61.7–74.5)	21.0 (19.0–27.0)	8.4 (7.8–9.0)	0.6 (0.5–0.8)
**Breakfast cereals**	Conventional	9	373 (371–415)	1.0 (0.9–14.0)	0.3 (0.3–6.0)	81.0 (65.0–82.0)	7.0 (6.0–21.0)	7.3 (7.2–8.0)	0.5 (0.4–1.5)
	Organic	9	375 (371–385)	1.0 (1.0–5.6)	0.3 (0.3–1.2)	81.0 (63.0–83.0)	6.1 (6.0–16.0)	8.0 (7.9–9.2)	0.1 (0.1–1.9)
**Snacks**	Conventional	6	383 (356–386)	14.0 (13.3–14.0)	2.5 (2.4–3.1)	59.2 (57.9–64.5)	33.5 (32.0–34.0)	5.4 (5.1–5.5)	0.6 (0.6–0.8)
	Organic	6	393 (381–403)	14.5 (12.8–15.2)	2.6 (2.6–3.1)	58.8 (55.1–64.1)	32.1 (31.0–35.0)	6.0 (5.9–6.1)	0.6 (0.6–0.8)
Bread and substitutes									
**Wraps**	Conventional	11	304 (303–326)	8.6 (8.6–9.6)	1.5 (1.5–2.4)	47.1 (47.0–48.9)	1.1 (1.1–1.5)b	8.1 (7.9–8.1)	1.6 (1.6–1.7)a
	Organic	11	309 (305–311)	9.2 (8.6–9.6)	1.6 (0.8–1.9)	47.0 (45.9–48.6)	1.6 (1.2–2.1)a	8.2 (7.9–8.7)	1.5 (1.4–1.5)b
**Crackers**	Conventional	10	429 (424–431)	11.5 (11.0–12.3)	1.4 (1.1–1.7)	66.8 (65.5–69.9)	2.0 (2.0–2.0)a	11.1 (11.0–2.0)	1.8 (1.8–2.0)
	Organic	10	427 (426–438)	12.2 (12.0–13.0)	1.9 (1.9–2.1)	65.1 (63.0–68.0)	1.9 (1.6–2.0)b	12.0 (11.1–13.0)	2.0 (1.4–2.0)
**Breadsticks**	Conventional	9	433 (415–451)	12.5 (8.0–16.0)	2.6 (1.3–4.1)	67.0 (62.3–71.0)	3.3 (1.9–3.5)	12.7 (12.2–13.0)	2.0 (1.8–2.1)
	Organic	9	436 (416–467)	11.6 (10.4–17.0)	2.0 (1.8–2.6)	65.0 (61.0–66.6)	2.4 (2.1–2.6)	13.0 (12.0–13.0)	1.9 (1.6–2.1)
**Bread**	Conventional	6	256 (252–268)	3.9 (3.3–4.6)	0.5 (0.4–0.7)	42.3 (32.0–46.1)	4.8 (4.6–4.9)	8.3 (8.2–8.6)	1.3 (1.2–1.5)
	Organic	6	254 (206–267)	4.2 (3.6–4.3)	0.5 (0.4–0.6)	44.8 (43.0–45.2)	3.1 (2.1–4.9)	9.0 (8.4–9.7)	1.3 (1.2–1.5)

**Rusks**	Conventional	6	399 (385–412)	7.6 (5.0–8.0)	1.0 (0.7–2.0)	72.1 (67.2–73.0)	7.0 (4.0–7.7)	10.4 (10.0–11.0)	1.2 (1.2–1.3)
	Organic	6	395 (384–406)	5.7 (4.0–6.8)	0.8 (0.5–1.1)	70.3 (67.0–72.0)	5.5 (3.5–6.7)	12.3 (11.7–12.5)	1.3 (0.6–1.3)
Pasta, rice and other cereals									
**Pasta**	Conventional	77	354 (351–359)a	1.5 (1.3–1.5)	0.3 (0.3–0.4)b	71.5 (69.2–73.1)a	3.0 (2.9–3.2)	12.7 (12.5–13.0)a	0.0 (0.0–0.0)a
	Organic	77	350 (347–354)b	1.5 (1.3–2.0)	0.4 (0.3–0.5)a	71.0 (66.6–71.0)b	3.2 (2.4–3.5)	11.5 (11.0–12.0)b	0.0 (0.0–0.0)b
**Rice and other cereals**	Conventional	12	354 (349–358)	1.2 (0.5–2.5)	0.2 (0.1–0.5)	70.5 (67.1–79.0)	0.4 (0.2–1.2)	9.3 (7.1–12.3)	0.0 (0.0–0.1)
	Organic	12	350 (344–354)	1.5 (0.9–2.2)	0.4 (0.3–0.5)	75.0 (67.8–78.0)	0.5 (0.2–1.1)	7.5 (7.0–9.4)	0.0 (0.0–0.0)
**Flour**	Conventional	12	339 (339–344)	1.0 (0.9–1.5)	0.2 (0.2–0.3)	72.0 (70.1–72.6)	1.0 (0.6–1.2)b	9.9 (9.0–10.8)	0.0 (0.0–0.0)
	Organic	12	345 (340–353)	1.0 (0.7–1.1)	0.2 (0.2–0.3)	72.5 (71.0–73.0)	1.7 (1.2–1.8)a	10.8 (9.8–11.0)	0.0 (0.0–0.0)
**Gnocchi**	Conventional	3	151 (123–156)	0.7 (0.4–1.4)	0.2 (0.0–0.2)	28.0 (24.5–32.6)	1.0 (0.1–4.7)	5.2 (3.5–5.4)	1.3 (1.3–1.4)
	Organic	3	126 (121–159)	0.6 (0.6–2.0)	0.2 (0.2–0.2)	25.2 (24.0–29.0)	4.0 (1.0–4.4)	3.6 (3.5–5.3)	1.3 (1.2–1.3)
Milk, dairy foods and plant-based drinks									
**Yogurt**	Conventional	46	96 (69–101)	3.4 (3.0–3.7)	2.4 (2.0–2.6)	12.9 (5.5–13.8)	12.0 (4.8–13.4)	3.6 (3.4–3.9)	0.1 (0.1–0.1)b
	Organic	46	96 (71–103)	3.3 (3.2–3.9)	2.2 (2.1–2.6)	12.1 (5.2–15.0)	11.1 (4.5–14.0)	3.6 (3.1–4.0)	0.1 (0.1–0.1)a
**Cheese**	Conventional	45	260 (216–285)	23.0 (16.0–28.0)	16.0 (11.1–18.7)	1.9 (1.0–2.8)	1.5 (0.3–2.3)	13.0 (8.0–17.0)	0.7 (0.6–0.8)
	Organic	45	260 (232–289)	22.0 (18.0–28.0)	15.8 (12.0–18.7)	2.0 (0.7–2.9)	1.3 (0.2–2.3)	14.0 (9.4–17.0)	0.7 (0.5–0.8)
**Milk**	Conventional	27	49 (47–65)	1.60 (1.6–3.6)	1.2 (1.1–2.6)	4.9 (4.9–5.0)	4.9 (4.9–5.0)	3.3 (3.2–3.4)	0.1 (0.1–0.1)
	Organic	27	48 (47–65)	1.60 (1.6–3.6)	1.1 (1.0–2.5)	4.9 (4.9–5.0)	4.9 (4.9–5.0)	3.2 (3.2–3.3)	0.1 (0.1–0.1)
**Plant-based drinks**	Conventional	5	44 (42–45)a	2.0 (1.8–2.1)	0.3 (0.3–0.4)	3.0 (2.6–3.3)a	2.5 (2.4–2.8)a	3.3 (3.0–3.5)	0.0 (0.0–0.1)
	Organic	5	39 (38–40)b	2.1 (2.0–2.1)	0.3 (0.3–0.4)	1.3 (0.9–1.6)b	0.8 (0.7–1.3)b	3.6 (3.5–3.8)	0.0 (0.0–0.1)

Juices, nectars and tea									
**Fruit juices and nectars**	Conventional	52	58 (54–60)	0.0 (0.0–0.0)	0.0 (0.0–0.0)	13.7 (12.5–14.3)	13.2 (10.7–14.0)	0.2 (0.1–0.4)	0.0 (0.0–0.0)
	Organic	52	57 (54–59)	0.0 (0.0–0.0)	0.0 (0.0–0.0)	13.3 (12.4–14.0)	13.0 (12.2–13.9)	0.2 (0.2–0.4)	0.0 (0.0–0.0)
**Ice tea**	Conventional	2	20 (19–20)	0.0 (0.0–0.0)	0.0 (0.0–0.0)	4.7 (4.6–4.8)	4.7 (4.6–4.8)	0.0 (0.0–0.0)	0.0 (0.0–0.0)
	Organic	2	2 (1–2)	0.0 (0.0–0.0)	0.0 (0.0–0.0)	0.1 (0.1–0.1)	0.1 (0.0–0.1)	0.0 (0.0–0.0)	0.0 (0.0–0.0)
Jams, chocolate spreads and honey									
**Jam and jelly**	Conventional	44	190 (187–211)a	0.1 (0.0–0.2)	0.0 (0.0–0.0)	45.0 (44.0–52.0)a	44.0 (43.0–50.5)a	0.4 (0.0–0.5)b	0.1 (0.0–0.1)
	Organic	44	166 (158–169)b	0.1 (0.0–0.3)	0.0 (0.0–0.0)	39.0 (37.5–40.0)b	36.0 (35.0–38.0)b	0.5 (0.3–0.6)a	0.1 (0.1–0.1)
**Honey**	Conventional	14	321 (320–326)	0.0 (0.0–0.0)	0.0 (0.0–0.0)	80.0 (80.0–80.0)	80.0 (80.0–80.0)	0.0 (0.0–0.4)	0.0 (0.0–0.0)
	Organic	14	322 (322–331)	0.0 (0.0–0.0)	0.0 (0.0–0.0)	80.0 (80.0–80.0)	80.0 (80.0–80.0)	0.6 (0.0–0.6)	0.0 (0.0–0.0)
**Chocolate spreads**	Conventional	3	525 (522–528)	29.0 (28.0–29.2)	10.0 (5.1–10.0)	59.0 (58.40–60.0)	58.0 (57.0–59.0)	5.4 (3.0–6.1)	0.1 (0.1–0.1)
	Organic	3	519 (519–542)	27.0 (26.8–31.4)	5.2 (5.2–8.6)	58.7 (54.4–59.0)	55.9 (51.6–56.0)	8.7 (8.5–8.7)	0.2 (0.2–0.2)
Fruit and vegetable-based foods									
**Tomato based-sauces**	Conventional	21	30 (27–59)	0.2 (0.1–2.7)	0.0 (0.0–0.4)	5.1 (4.3–6.0)	4.0 (3.7–4.5)	1.4 (1.2–1.7)	0.4 (0.0–1.0)
	Organic	21	31 (27–66)	0.2 (0.1–3.4)	0.0 (0.0–0.6)	5.5 (4.5–6.3)	3.9 (3.6–4.4)	1.3 (1.2–1.6)	0.1 (0.0–0.8)
**Dried fruit**	Conventional	15	584 (328–614)	48.0 (0.5–53.0)	3.9 (0.0–5.6)	12.0 (8.8–72.0)	4.6 (3.9–59.2)	18.1 (1.9–21.0)	0.1 (0.0–0.1)
	Organic	15	585 (340–627)	48.0 (0.5–55.0)	4.6 (0.1–5.5)	12.0 (5.1–72.0)	4.5 (3.7–59.0)	18.0 (2.4–21.0)	0.0 (0.0–0.0)
**Frozen vegetables**	Conventional	15	38 (33–56)	0.5 (0.3–0.7)	0.1 (0.0–0.1)	5.0 (2.9–8.2)	1.7 (0.4–2.5)	2.9 (2.7–3.5)	0.1 (0.0–0.1)
	Organic	15	35 (30–56)	0.4 (0.3–0.7)	0.0 (0.0–0.1)	4.5 (2.8–7.6)	0.5 (0.2–2.8)	3.0 (2.2–3.4)	0.1 (0.0–0.1)

Legumes									
**Dried legumes**	Conventional	21	332 (309–347)	1.6 (1.2–2.0)	0.3 (0.1–0.4)	46.9 (37.0–51.8)	2.5 (1.8–3.7)	22.0 (19.5–23.6)	0.3 (0.2–0.5)
	Organic	21	316 (292–355)	1.6 (1.0–2.5)	0.2 (0.1–0.5)	45.4 (41.7–49.3)	2.4 (1.3–3.5)	21.0 (20.0–23.0)	0.2 (0.1–0.3)
**Canned and frozen legumes**	Conventional	34	95 (83–112)	0.5 (0.5–1.8)	0.1 (0.1–0.3)	13.2 (12.1–15.8)	0.7 (0.6–1.0)	6.2 (5.5–7.0)	0.8 (0.7–1.0)
	Organic	34	92 (80–97)	0.6 (0.4–1.7)	0.1 (0.1–0.2)	12.9 (10.8–14.2)	0.7 (0.3–1.3)	6.2 (5.4–6.9)	0.7 (0.6–0.9)
Oils, fats and dressings									
**Olive oil and other vegetable oils**	Conventional	32	824 (822–827)	91.8 (91.6–92.0)	13.5 (13.0–14.1)	0.0 (0.0–0.0)	0.0 (0.0–0.0)	0.0 (0.0–0.0)	0.0 (0.0–0.0)
	Organic	32	824 (824–828)	91.6 (91.6–92.0)	13.9 (13.0–14.0)	0.0 (0.0–0.0)	0.0 (0.0–0.0)	0.0 (0.0–0.0)	0.0 (0.0–0.0)
**Animal fats and margarine**	Conventional	10	752 (747–754)	83.0 (83.0–83.0)	57.6 (53.0–58.0)	0.4 (0.0–0.7)	0.3 (0.0–0.7)	0.5 (0.0–0.6)	0.0 (0.0–0.1)
	Organic	10	750 (739–754)	83.0 (82.0–83.0)	58.0 (56.0–59.2)	0.1 (0.0–0.7)	0.1 (0.0–0.7)	0.2 (0.0–0.6)	0.0 (0.0–0.0)
**Vinegar**	Conventional	9	25 (17–83)	0.0 (0.0–0.0)	0.0 (0.0–0.0)	0.5 (0.5–16.0)	0.5 (0.5–16.0)	0.1 (0.1–0.1)	0.0 (0.0–0.1)
	Organic	9	25 (17–178)	0.0 (0.0–0.0)	0.0 (0.0–0.0)	0.5 (0.1–39.0)	0.5 (0.1–39.0)	0.1 (0.1–0.1)	0.0 (0.0–0.0)

Values are expressed as median (25th–75th percentile). For each category, different letters in the same column indicate significant differences among conventional and organic products (Mann–Whitney non-parametric test for two independent samples), $p < 0.05$.
